# Making kangaroos grievable; making grievability non-human

**DOI:** 10.1007/s40656-022-00494-y

**Published:** 2022-04-19

**Authors:** Yvette Kim Clarissa Wijnandts

**Affiliations:** History Department, The University of Adelaide, Adelaide, SA Australia

**Keywords:** Food animals, Kangaroos, Grievability, Commercialization, Discourse

## Abstract

When Australian economist Ross Garnaut proposed to increase the commercial kangaroo industry in 2008, it started a national debate on the supposed edibility of kangaroos. Campaigns against the commercial kangaroo industry and hesitance amongst many consumers to eat kangaroo reflect concerns about viewing kangaroos as food. This article explores the reactions and challenges that originate from the kangaroo’s changing role in society by using Judith Butler’s concept of *grievable lives*. Using this framework shows that what animals we eat goes beyond nutritional value; it symbolizes deeper values regarding human–animal relations and illustrates why and how not all animals are seen and treated as the same.

## Introduction

This paper explores the backlash against the proposed role of the kangaroo as a food animal in Australia. The role of kangaroos in Australian society has circulated between being a national symbol, a pest, and food animal. On the one hand, kangaroos are a national symbol. Skippy, the character in the world-famous children's series about the adventures of a boy and his pet kangaroo, was widely popular and loved in and outside of Australia (Burnstock, n.d.). At the same time, and this is probably less-known amongst non-Australians, Australians see kangaroos also to a certain extent as pests^1^ and as a food source.

Kangaroos have been a food source for thousands of years in Australia. Aboriginal peoples have hunted kangaroos for their meat, skins, and symbolic value (Thomson et al., 2006). Most Aboriginal kangaroo hunting has been for private use; the kangaroos were consumed by the hunters, their families, and their tribes. Since the early 2000s, the South Australian government has attempted to involve Aboriginal peoples in the commercial kangaroo industry as well (Thomson et al., 2006). But Aboriginal peoples are not the only Australians who hunt and eat kangaroos. In the nineteenth century, settler Australians started with the export of kangaroo products to Europe and the US (Hercock & Tonts, 2004). The main product exported was kangaroo skins, an industry that grew from 320 skins exported in 1843 to 135,000 skins in 1892 and, a century later, 1,614,705 skins in 1992 (Hercock & Tonts, 2004, p. 218).

However, practices of hunting, consuming, and trading in kangaroos do not go uncontested in Australia despite their long histories. National campaigns against the commercial kangaroo industry and a general hesitance of non-Aboriginal and Torres Strait Islander consumers reflect a reluctance to see kangaroos as food. Originally, the lack of availability of kangaroo meat was an important factor in consumers’ choices to not eat kangaroos. In New South Wales, Queensland, and Victoria, state legislation did not allow selling kangaroo meat for human consumption until 1993, while the South Australian government was an early exception by never placing a ban on the sale of kangaroo meat for human consumption (Waitt, 2014). Only since the year 2000 has kangaroo meat been widely commercially available in supermarkets and butcher stores (Waitt, 2014; Waitt & Appleby, 2014). Now that kangaroo is more readily available in Australian supermarkets, research has shown that other factors continue to contribute to low consumer numbers. Kangaroo meat is often seen as pet food, rather than food for humans. It is also hard to prepare due to its low levels of fat which leads to kangaroo meat easily being overcooked and therefore becoming tough and hard to chew and swallow (Waitt, 2014). Finally, there are also sentimental reasons for low kangaroo consumption rates. Kangaroos have long been a national symbol in Australia. They appear on the coat of arms. Kangaroos are also part of many stories children grew up with (Morton, 1990; Simons, 2013), and have played a beloved television character in the hugely popular television show *Skippy*, mentioned above (Burnstock, n.d.; Hercock & Tonts, 2004; Simons, 2013; Waitt, 2014). In other words, the role of kangaroos in Australia is complex and various.^2^

This paper explores how opponents of the kangaroo industry approach the topic of the commercial kangaroo industry. While Aboriginal and Torres Strait Islander peoples used kangaroos as a food source for thousands of years, this paper will focus on non-Indigenous discourses on the role of kangaroos as food animals because kangaroo is a relatively new food animal for non-Indigenous Australians. This article has two aims. First, it presents a discourse analysis of the opponents of the kangaroo industry, specifically Animals Australia and Voiceless. Discourse analysis will show how these stakeholders contribute to the production and transmission of meaning, and how “institutional roles are contradicted and power relations developed and maintained” (Wodak, 2000, p. 185). Following this, it explores how Judith Butler’s concept of *grievable lives* can be applied to these conflicting discourses. Applying the framework of *grievable lives* to the practice of eating kangaroos will allow a deeper understanding of why this practice is more contentious than the eating of other food animals.

The analysis I present is not seeking to challenge those critical of the industry, but instead examines how they frame their arguments. Doing so helps to shed light on how our beliefs are predicated on more than just whether it is good or bad to eat certain animals. It explores why some animals are deemed to be exceptional or should be treated differently from others. I believe that examining these occurrences further will allow a deeper understanding of how food choices, especially food derived from animal bodies, are made within broader contexts.

Research material is limited to public discourse by opponents of the industry. I conducted a digital content analysis to obtain and analyze data. First, I formulated research questions to examine how practices of eating kangaroos are discussed in public discourse. I then selected a sample. For the purpose of this paper, I based the analysis on the websites of two animal rights groups. The reason for focusing on Voiceless and Animals Australia is that they are both animal welfare organizations that are based in Australia, and include kangaroos as one of their issues of concern within a broader, general focus. Voiceless is particularly interesting for its connections to THINKK.^3^ Animals Australia is one of Australia’s largest animal protection organizations.^4^ I focused on their pages on kangaroos in the stage of drawing a sample for analysis. I then defined three main themes by closely engaging with the content on the web pages. Finally, I analyzed and interpreted the data by identifying several key themes in the text and images on the webpages.^5^ I will first elaborate on the concept of grievability and how I use it to explore the campaigns against the kangaroo industry. I will then explain why the topic of kangaroos offers a useful case study to think about food animals, and why certain animals are deemed to be suited as food animals more or less than others. Thirdly, I will explore three themes that arose from the websites that illustrate how kangaroos fit within the framework of grievability. Finally, I will explore how this project can have value for broader analyses of human–animal relations.

## Grievability

This argument draws on Butler’s concept of grievable lives to explore further underlying drivers for the arguments presented by animal welfare groups opposing the kangaroo industry. Applying Butler’s framework of *grievable lives* will offer a critical approach to the arguments presented by these groups.

Butler (2009) expands on the concept of grievable lives in their book *Frames of War*. The work consists of five essays on contemporary wars and an introduction in which Butler explains their key concepts. Butler aims to understand why groups of people in the “West”, with a focus on the US, are ready to accept, and at times even embrace, war horrors suffered by those in the Middle East. Critiques have been made about Butler’s approach being too anthropocentric in their use and understanding of the concept.^6^ Whereas I agree that Butler uses anthropocentric language, I do not believe that their theory is inherently anthropocentric. By applying Butler's concept of grievable lives to kangaroos in Australia, theory will not only help make this specific issue understandable but kangaroos will also be used to gain a deeper understanding of the theory.

Butler uses the concept of grievable lives to explain that grief is produced by mechanisms of power situated in social and political structures. Butler’s concept of grievability offers a tool to explore why the proposition to increase kangaroos’ role as a food animal drew different responses. There are several reasons for choosing Butler’s particular concept of *grievability* to examine the contentiousness of kangaroos as food. First, most theories so far have focused on whether something is or is not ethical when it comes to animal consumption. Yet, as Cora Diamond (1978) argued in her iconic paper "Eating meat and eating people":

This is a totally wrong way of beginning the discussion, because it ignores certain quite central facts-facts which, if attended to, would make it clear that rights are not what is crucial. We do not eat our dead, even when they have died in automobile accidents or been struck by lightning, and their flesh might be first class. (...) Now the fact that we do not eat our dead is not a consequence-not a direct one in any event-of our unwillingness to kill people for food or other purposes (p. 467).

In other words, eating animals, or eating *certain* animals, is not just a consequence of rational and moral arguments, like animal scholars such as Peter Singer (1975) and Tom Regan (Regan & Singer, 1976) argue. Food is not only about the morals of ending the life of the animal that is to be consumed. Rather, food is about a willingness to eat certain things/beings and not others.

Other scholars have used Butler’s concept of grievable lives to look at non-human animals. For example, in "Pet grief: when is non-human life grievable?", David Redmalm (2015) identifies three key factors that determine grievability of a life based on Butler’s work. Redmalm proposes that the grieved life has to be irreplaceable, the grief has to be "transformative and [take] unpredictable expressions" (p. 20), and since the death is of an *embodied* person, the grief has to be physical too. Redmalm's approach to grievability is useful for his research topic, which is pets with whom people form individual connections. However, in this research, the emphasis lays more on kangaroos as a species rather than individuals. Therefore, I picked the requirements from Butler's approach of grievability of populations. I acknowledge that a species-level approach may distract from the lived realities of individual kangaroos. However, I also believe that in practices of eating kangaroos and in the discourses surrounding this, arguments for and against are based around kangaroos as a species, rather than narratives of individual kangaroos.

While only referencing Butler’s work on grievability briefly (Dooren, 2014, p. 142), Thom van Dooren also explores phenomena such as grief, death, and mourning in non-human animals. Using the case study of the extinct in the wild Hawaiian crow, he illustrates how humans are not the only species that mourn and uses examples of grieving animals to debunk the history in Western thought of understanding death as something that sets humans apart from all other animals. More so, Van Dooren argues that grief and mourning help (certain) animals, human but also non-human, to adapt to the changed realities of the world as it changes with the loss of others. Grief and mourning allow for the acknowledgement that the world is a shared space.

Australian writer and Associate Professor in English David Brooks (2020) explores the possibility of kangaroos’ grief of their death in “The grieving kangaroo photograph revised.” Discussing grief outside of Butler’s framework, but with a focus on the experience of kangaroos themselves. He discusses a 2016 photograph that received media attention. Firstly, because the photo was understood to portray a dying female kangaroo surrounded by a grieving male kangaroo and her Joe. However, shortly after its original publication, the photograph drew attention for a second time; various kangaroo experts argued that the motivations of the male kangaroo were centered around territorial and reproductive urges of the male kangaroo rather than grief. Brooks argues that these experts may have been wrong and that, more importantly, denying non-human animals grief and non-normative expressions of grief (“normative” meaning “accepted by humans”) “the experts [are] providing us, ironically (and yet again), with an example of the self-same anthropomorphism of which they accuse others” (2020, p. 207). Brooks’ argument shows that relationships and understanding between humans and kangaroos are not only complex but also multi-directional.

In my use of *grievability*, there are two main requirements. First, the importance of kangaroos *as lives that should be led* and should not be made to end, is central. In *Frames of War*, Butler (2009) explains that grief is about a life that has ended. *Grievability* places the future anterior of a life central. In other words, a grievable life is a life that while it is still being lived, is seen as valuable *and* as a life that should not end prematurely. Second, to be seen as grievable, a life has to be *recognized* as part of an (imaginary) shared identity of precarity. This shared identity is always imaginary, as all lives are precarious. All life will end after all. The finiteness of our lives means our lives always lie in the hands of others. Precarity and grievability, according to Butler, distinguish those we see as possible threats to our lives and those who we recognize to be in the same situation as us. We have obligations towards those with who we share precarity (2009, pp. 13–15). At the same time, we see Others as threats to our lives, as this is part of living finite lives. Framing is key in Butler’s understanding of grievable lives. Who is included in “we” is highly contextual and determined by frames of grievability.^7^ Whether and to what extent a life is grievable varies between peoples and, as I argue, species. It should be underlined that grievability is not inherent to a person or species. Instead, it is a tool to understand why and how a life is understood. Butler does *not* argue that the lives of Palestinians are not grievable, nor do I want to argue that the lives of certain animals should be less grievable than others. Instead, grievability is a framework and tool to understand why certain lives are deemed to be more important than others as well as a critique on the treatment of those who are deemed to be less grievable than others.

This research focuses not on whether kangaroos should be food animals or not, but why this is a question while certain other animals are generally accepted as foods and others less. What makes Butler's work on grievability valuable for the critical analysis of our perception of animals is that it allows an understanding of difference on different levels, or, in Butler's words, within different frameworks. All lives will end but only some are seen to be worthy of protection from premature death. Who is included and excluded in these frameworks is dependent on wider cultural norms that have material effects as lives literally depend on it, whether these lives are human or non-human. At the same time, these frameworks are arbitrary in that inclusion and exclusion could change as norms and values are always changing.

## Why kangaroos?

In 2008, Ross Garnaut, an esteemed economics professor, recommended greatly expanding the kangaroo market to cut down the sheep and cattle industry as part of long-term goals to reduce Australia's carbon emissions (Peace, 2011, p. 80). This proposal was part of a bigger project, namely the Garnaut Climate Change Review. This Review aimed to explore how climate change would affect the Australian economy and find strategies to improve the prospects for economic and environmental sustainability (Garnaut, 2011).

Whereas the media attention for Garnaut's proposal to reduce carbon emissions disappeared almost as fast as it arose, his proposal to make kangaroo a staple food in the Australian diet gained much more media attention. While there is a long history of eating kangaroos in Australia, kangaroos were not seen as an obvious food source by non-Indigenous Australians when Garnaut’s review was published.^8^ The Kangaroo Industry Association of Australian [KIAA] was one of the first to react and used Garnaut's claims about the environmental benefits of kangaroo meat to promote their industry (Peace, 2011). Yet, Garnaut's proposal also faced a lot of rejection for several reasons such as taste and unwillingness to reduce carbon emissions in general, arguing that Australia played a minor role in worldwide global warming (Peace, 2011). Next to this, Garnaut's ideas on the benefits of kangaroo hunting were critiqued as well.

The main argument against Garnaut’s proposal that greater kangaroo consumption could have environmental benefits is that there has been no significant reduction in the Australian sheep industry following the growth of the kangaroo industry. This argument has been made in the 2011 THINKK report (Ben‐Ami et al., 2011).^9^ At the same time, the increased commercial kangaroo industry has led to an increase in the suffering of animals (in this case the kangaroos) that is not reasonable considering the limited benefits that follow from this increase. The report gives five reasons for this. The first is that a large number of joeys will be the collateral kill of kangaroo hunting. Next to this, miss-shot kangaroos will suffer from injuries. Third, the genetic diversity of the kangaroos will decline long-term. Fourth, the Code for commercial kangaroo shooting is not always followed.^10^ Finally, public attitudes towards hunting are driven by three key factors, namely commercial value, “pest” status, and ecological concern. Both Voiceless and Animals Australia base a large part of their campaigns on the results of this study. However, THINKK’s research has not gone without being scrutinized either. In response to the report, Environmental scholars Rosie Cooney, Alex Baumer, Peter Ampt, and Michael Archer (2012) published the chapter “THINKK again: getting the facts straight on kangaroo harvesting and conservation” in a book with the title *Science under siege: zoology under threat* in response to the 2011 THINKK report. The focus of the chapter is, a^2012^ccording to the authors, “the identification and correction of what, in [their] view, are major flaws and inaccuracies of fact and reasoning, rather than debating an alternative vision for kangaroo management [in the THINKK report]” (Cooney et al., 2009, p. 151). THINKK later responded again but the groups did not reach a consensus on the value, environmental, ethical, and economic, of the kangaroo industry.

Despite the debate over how beneficial increasing the commercial kangaroo industry would be, the industry has now grown to be the biggest commercial wildlife industry in the world. Even then, though, there is disagreement about how large the industry is.^11^ In Australia itself, the kangaroo industry has predominantly been a pet food industry since the 1940s. Up until today, even though kangaroo meat is easily available, the consumption of kangaroo meat is marginal compared to the consumption of pork, beef, lamb, veal, and chicken.^12^ Research has shown that the main reasons given for this by consumers are quite paradoxical. On the one hand, kangaroo meat is seen as unsuitable for human consumption because kangaroos are seen as pests and their meat is associated with pet food. On the other hand, it is the kangaroo's status as a national symbol that makes them seen as unsuitable for human consumption. In other words, kangaroos should not be eaten because they are, in a sense, too special to be food (Waitt, 2014).

Kangaroo hunting is strongly regulated. In Australia, rules and regulations concerning hunting differ based on different animal species, and in particular between feral and native species. Feral animals are animals that have escaped captivity and/or domestication to survive in the wild (Donaldson & Kymlicka, 2011, p. 224). In general, they are perceived as a threat to the native flora and fauna in Australia. Whereas hunting feral species generally requires only a basic hunting permit, all hunting of native species, like the hunting of kangaroos, is illegal or at least heavily controlled, depending on the states' regulations (Finch et al., 2014, p. 77).^13^ Hunting kangaroos for commercial purposes is legal in five states in Australia namely New South Wales, Queensland, South Australia, Tasmania, and Western Australia. This means that commercial kangaroo hunting is allowed on over 75% of Australia’s surface. All five states state clearly that wildlife management is central in the regulations concerning commercial kangaroo harvesting (Government of South Australia, 2020; Government of Western Australia, n.d.; NSW Department of Planning, 2021; Queensland Government, 2021). Also, in all five states, commercial kangaroo harvesters must follow the *National codes of practice (commercial and non-commercial) for the humane shooting of kangaroos and wallabies* (Natural Resource Management Ministerial Council, 2008). The code offers explicit guidelines as to how the specific killing part of kangaroo hunting should take place to secure minimum suffering for the animals and their young. Despite being arguably the most regulated industry, it also faces the most backlash.

This article takes a deeper look at the arguments regarding the suitability of kangaroos as food animals as presented by Voiceless, the largest animal rights group in Australia, and Animals Australia. Extensive research has been done on whether some animals should be food or not for moral, economic, and pragmatic reasons (such as the Garnaut review), but significantly less into why we deem certain animals as foods and others not, even if scientific evidence shows that certain forms of animal consumption would be more desirable than others.^14^ This article uses the case study of the kangaroo to explore various discourses that contribute to the suitability of certain animals as food animals.

## Kangaroos as grievable non-humans

While replacing the consumption of farmed meat with the consumption of kangaroo meat may possibly have benefits for the Australian landscape and environment, the suggestion to implement this change has not been taken up without pushback. This section focuses on three themes that emerged from analysing the websites of Voiceless and Animals Australia, namely the focus on the role of kangaroos as food animals, the language used in opposing discourses presented by the organisations, and the emphasis on environmental management in narratives about kangaroos. I will then use Butler’s concept of *Grievability* to explore how, within these three themes, kangaroos fit within her framework of grievable lives in these discourses.

### Framing kangaroos

Both Voiceless and Animals Australia oppose different practices related to the meat industry in Australia, focusing on industrial farming and live export. The website of Animals Australia opposes other practices of hunting both domestically and internationally. Yet, on the Voiceless website, hunting animals other than kangaroos is hardly addressed, showing that there is something particular about the kangaroo compared to other animals that can be hunted in Australia. At the time that data for this article was collected, five pages on the Voiceless website carry the “hunt” or “hunting” tag. One discusses duck shooting, one discusses animal law in South Africa, and one briefly references seal hunting in Canada and compares seal hunting to kangaroo hunting in Australia. In addition, there is a page dedicated to problems surrounding the fishing industry. The emphasis on kangaroo hunting over other animals that are being hunted does *not* mean that the founders, directors, and supporters of Voiceless *support* hunting other animals. Rather, it underlines their concern regarding *kangaroo* hunting.

The emphasis on kangaroos over other hunted animals in the Voiceless campaigns reiterates how kangaroo lives are more grievable than other food animals’ lives. It should be re-emphasized that Australian law already protects most wild native animals in Australia but this protection does not extend to feral species. This indicates that the campaign against commercial kangaroo hunting is about something *more* than the harm kangaroos suffer from being killed. Even if everyone would agree with Voiceless' arguments against kangaroo hunting, it cannot be denied that other hunted (food) animals in Australia, such as deer and hogs, suffer the same fate. For example, feral hogs are regularly killed, sometimes for food purposes, but cause less financial damage than kangaroos, according to the Pest Animal Cooperative Research Centre (Gong et. al. n.d.). As mentioned before, within Australian law, the hunting of kangaroos is the most regulated. All feral animals can be hunted with easily accessible basic hunting permits. Also, if kangaroos suffer from physical harm due to hunting, this must certainly be true for feral hunted animals too, since there are less strict codes and regulations for these hunting practices than for the hunting of kangaroos. Furthermore, while other animals may take care of their young differently than kangaroos, killing mother animals will likely lead to suffering for their young. Despite kangaroos’ suffering and wellbeing being overly represented in animal welfare campaigning over other food animals, including other game animals, it remains unclear from a moral perspective as to why specifically *kangaroos* should not be hunted.

### Language

While Butler only briefly touches upon language in the construction of grievable lives, campaigns surrounding the role of the kangaroo as a food animal show how language can contribute to the grievability of human and non-human animals. The relationship between language and human power over non-human animals has been examined previously by linguistic scholars. English scholar Stibbe (2001) for example illustrates how language at all levels contributes to masking the harm humans cause non-human animals. Individual words play an important role here—for example, terms such as “meat” and “product” are used instead of bodies when talking about animal flesh that is sold for commercial purposes—but also grammar. Language thus contributes to producing and maintaining oppression. Adding to Stibbe’s argument, Jepson (2008), also located in English language studies, illustrates how the connotations of certain words related to death carry heavier weight when used in regard to human death than when used to describe non-human deaths at the hands of humans.

While language in regard to animal deaths is usually used in a way to minimize the harm associated with death, the opposite is happening in the campaigns against the commercial kangaroo industry. The language used to describe kangaroo’s deaths caused by the kangaroo industry clearly aims to show suffering. For example, kangaroos are "killed" and “shot” rather than "slaughtered" or "culled" (Animals Australia, n.d.-b). This word choice is evocative and equates animal to human suffering. This implies that their deaths are unfair, rather than necessary or pragmatic. Also, significant emphasis is placed on the fate of Joeys, young kangaroos (Animals Australia, n.d.-b). They will be killed too, most likely in a cruel manner, or left to starve. Voiceless follows similar trends and also actively addresses the importance of language when discussing kangaroos. For example, they state that “Animal protection advocates also raise broader issues with the use of the term ‘pest’ to describe any sentient animals, as it suggests that the lives of these animals are inherently less valuable than others” and “Voiceless and other animal protection advocates challenge these ways of conceptualising kangaroos, questioning their classifications as ‘resources’ and ‘pests’” (Voiceless, n.d.).

Using language that underlines, rather than denies suffering, contributes to constructing kangaroo lives as grievable lives. First, by using the same language for kangaroo deaths as would be used for human deaths, or at least human deaths that are grieved, kangaroo lives become relatable. If kangaroo deaths are not fundamentally different from human deaths, and neither is the value of their lives, they are fitted within a framework of grievability. This language also conveys the *importance* of their lives. If their lives end prematurely, this is not something to accept but something to grieve because these kangaroos still had a life ahead of them.

### Pastoral care narratives

Both Voiceless and Animals Australia emphasise how kangaroos are part of the Australian landscape. The main page on Voiceless’ website about kangaroos starts with the statement that “[k]angaroos are native animals who have adapted uniquely to the Australian landscape. Their ancient ancestor has been traced back 24 million years to the Palaeopotorous—the starting point of all known kangaroo species”, underlining how they are part of the Australian landscape. Similarly, the Animals Australia page on kangaroos starts with the following text: “Each night in remote areas of the Australian outback, thousands of kangaroos graze peacefully, stand up on hearing an approaching vehicle, stare into a blinding spotlight, and are shot for their flesh and skins.” A little further, the “Australian bush” is referenced, and the reader is warned that “we may face a future where this iconic native animal is nothing but a memory” (Animals Australia, n.d.-b).

In Australia, native nature has come to play a central part in nationalist discourses. This has not always been the case. During early settlement, native Australian animals were seen as inferior to European animals. This had multiple negative consequences for them, such as the active eradication of native species as well the lack of active preservation when their survival was under threat because of newly introduced European species. Around the end of the nineteenth century, a shift took place toward valuing the Australian landscape and its (non-human) inhabitants. During this period, Australian settlers had already been living on the Australian land for multiple generations. Australian nature became a site of national symbolism and conservation ideals were inextricably linked to ideas of good Australian citizenship (Franklin, 2011). Migrants, and refugees especially, have often been seen as a threat to Australian identity, just as feral animals are positioned as threatening to the unique and, arguably, vulnerable native Australian nature (Gressier, 2016, p. 53). The “responsibility” taken up by the predominately white Australian settlers to preserve the native Australian landscape, and its relations to constructing Australian identity and belonging based on this, has been analyzed extensively (Franklin, 2011; Smith, 1999).

Somewhat ironically, the supposed importance of kangaroo management for the purpose of conservationism has increasingly turned into a reason for consumers to buy and eat kangaroo meat. One of the more notable developments regarding this is the emergence of so-called “kangatarianism”.^15^ A kangatarian can be defined as “someone who chooses not to eat fish or meat except the meat from a kangaroo” (Maxwell, 2011). In general, kangatarians identify with ethical vegetarians, believing that eating kangaroo meat is an environmentally friendly way of consuming meat, in which animal harm is reduced to a minimum. There remains a lack of academic research towards kangatarians and there is no scientific data on when kangatarianism arose and how many people identify with this lifestyle.^16^ While the environmental impact of eating meat is increasingly acknowledged and problematized on a global scale, kangaroo meat is a little more complicated. Whether the consumption of kangaroo meat is less damaging to the Australian landscape than the consumption of other animal bodies or not, the kangaroo is inherently part of the Australian landscape that is deemed to be in “need” of governance and protection.

These conservationist aspects in debates surrounding the role of kangaroos as food animals can be understood to contribute to constructions of grievability surrounding kangaroo lives. Firstly, campaigns for and against the commercial kangaroo industry both agree conservationism is a worthwhile cause and thus help construct the narrative of the Australian environment to be precarious. Kangaroos are part of this shared precarity. This shared identity fits in a long history of being deemed precarious and of “Australians” “needing” to preserve it (Simons, 2013). However, it should be emphasised that while Animals Australia and Voiceless draw from and tap into nationalist discourses that have a long history, they do *not* appear to reify their original white nationalism. Opponents of the kangaroo industry specifically underline that kangaroo lives should not be sacrificed to ‘protect’ the landscape.

## Conclusion

Derrida (2002) made the argument that when thinking about “The Animal”, it is critical to recognize the plurality of this category. “The Animal” does not exist, just as “The Human” does not exist; material and conceptual differences lead to radically different experiences. This article has discussed the challenges and reactions to the circulating role of the kangaroo within Australian society. Rather than fitting within one category of animal, kangaroos destabilize the different relationship categories humans engage in with non-human animals; kangaroos inhabit multiple categories simultaneously, being an animal that is loved as a national symbol, a pest for farmers, and becoming increasingly an animal to be eaten.

Again, the aim of this article was not to argue that campaigning against kangaroo hunting is or is not beneficial but rather to examine why and how campaigns against their hunt take place and how these discourses, albeit unintentionally, feed into the grievability of these animals. This paper used a Butlerian framework of grievablity to understand how kangaroos are presented by opponents of the commercial kangaroo industry. Grievability, in this paper, requires lives to be represented as lives that should be led, and thus should not be allowed to end prematurely, as well as lives that belong in an (imaginary) shared identity of precarity. The portrayal of kangaroos on the websites of Voiceless and Animals Australia meets these criteria. While many animals die in similar circumstances as kangaroos, the emphasis on the unfairness of *kangaroos* dying, implies their lives are meant to be led. The language used to critique the kangaroo industry does not only condemn the untimely deaths of kangaroos, but the difference between the values of lives between the species is also denied by using the same language that would be used to describe human–animal lives and deaths. Finally, a narrative that is often drawn upon in regard to kangaroos, is that of conservationism. It is in this narrative that role of kangaroos in the shred precarity of the Australian landscape, and Australia itself, is re-emphasized. Thus, whereas grief may be a human emotion, this does not mean that one has to be human to be grievable.

I have focused on the websites of Voiceless and Animals Australia to explore the grievability of kangaroos. I acknowledge that this is only a small part of the broader discourse surrounding the role of kangaroos as food animals and is therefore of limited value. Yet, the analyzed campaigns against the commercial kangaroo industry bring attention to suffering and contribute to public discourse. In addition, previous research has shown that similar sentiments are present among meat consumers and that many Australians who eat beef, pork, or chicken feel differently about eating kangaroo due to affective reasons (Craw, 2008; Waitt, 2014). In the case of the kangaroo, food is not just about nutrition or moral argument; broader values are involved. While grievability is to a certain extent arbitrary, grievability shapes, but is also shaped by, pre-existing knowledges in the broadest sense of the word. It is through representation that lives become visible as precarious. Media plays a role in this because it is only when a life is visible that we can start to perceive it as a life (Butler, 2009). Further research on the discourses surrounding the kangaroo industry would be valuable as the case study is multi-faceted, complex, and has not been researched extensively yet from a sociological or STS perspective.

I have utilized the framework of grievability on the cases presented by Voiceless and Animals Australia to explore not only what arguments they present against the kangaroo industry, but also to analyse deeper why the kangaroo industry is such a controversial one, and how concern about this industry and the affected animals is conserved. Whether animals are deemed to be edible or not can depend on other factors than species alone; the role of the kangaroo as a food animal specifically may have fueled the conversation around kangaroos’ lives and deaths more so than when their bodies were used for pet food, for example. In addition, factors such as geographical closeness to humans, physical and/or emotional similarity to humans, or even ‘cuteness’ could contribute to animals being perceived as part of a community and a life worth protecting. While grievability may not be the only deciding factor as to what makes certain animals edible or not, I argue it is an important one. Not only can the framework of grievability offer insights as to what contributes to closer human–animal relations but a greater understanding of why humans care about animals can also help inform strategies to change relations between humans and certain animal species.

## Footnotes

1 The kangaroo population has arguably grown in numbers since European settlement. Most Australians have been, or at least know someone who has been in a car accident involving kangaroos during or after sunset. In addition, kangaroos are often blamed for competing over food with livestock (O’Grady, 2018).

2 Human–animal relations psychology scholar Hal Herzog (2011) explored the different relationships humans engage in with animals. Herzog identified three main roles humans tend to categorise animals in namely food, companions or collaborators, and pests. The kangaroo, coincidentally, occupies all three spaces and thereby can be seen as a support to Herzog’s claim that “the flagrant moral incoherence [described in his book] … are not anomalies or hypocrisies. Rather, they are inevitable. And they show we are human” (p. 279).

3 THINKK is the Think Tank for Kangaroos that is part of the University of Technology Sydney and collaborates with the Institute for Sustainable Futures. Voiceless is one of its key supporters (THINKK [The Think Tank for Kangaroos], n.d.).

4 According to Animals Australia, they are “Australia's leading animal protection organisation” (Animals Australia, n.d.-a). While no sources beyond Animals Australia themselves support or refuse this claim, Animals Australia is a not-for-profit body that represents a has more than 2 million individual supporters and represents approximately 40 member groups (Mummery & Rodan, 2019, p. 59).

5 I acknowledge that it is best practice to identify categories and themes with more than one researcher to check reliability (McMillan, 2000, p. 80). However, as the research of this paper is part of a postgraduate research project, I am expected to conduct the research by myself and lack the resources to bring in extra researchers.

6 See for example Carey Wolfe's argument that "Butler's effort (…) runs aground on the question of nonhuman animals" (2013, p. 18).

7 Butler generally identifies “a community of belonging on the basis of nation, territory, language, or culture” (2009, p. 36) for the purpose of her own argument.

8 There are no clear numbers on how much kangaroo is consumed on a yearly basis. Waitt (2014) explains how research suggests that in 2007 only 4.7 percent of Australian households eat kangaroo at least once a month despite it being easily available in supermarkets (p. 407). Also, most kangaroo consumption happens in restaurants rather than at home, underlining how it is not a staple meat (ibid).

9 In 2011, THINKK published "The ends and means of the commercial kangaroo industry: an ecological, legal and comparative analysis" to criticise the practices of the commercial kangaroo industry in Australia.

10 Every commercial kangaroo hunter is obliged to follow the National Code of Practice for the Humane Shooting of Kangaroos and Wallabies for Commercial Purposes (Natural Resource Management Ministerial Council, 2008).

11 In 2005 the industry was estimated to bring in over 200 million Australian dollars per year and provide jobs for about 4000 people (Kelly, 2005). Yet, in their report on the kangaroo industry, THINKK argues that this is an extremely generous estimation and the true income from the industry would be much closer to 50–60 million dollars per year (Ben‐Ami et al., 2011).

12 Australians ate an average of approximately 110 kg of beef, pork, poultry, sheep, and fish combined per person per year in 2018, making Australia the second-most meat consuming country per person in the world (Whitnall & Pitts, 2020).

13 Exceptions apply for Aboriginal people. Under the National Parks and Wildlife Conservation Act 1975 it is provided that Aboriginal people in Australia can "continu[e] in accordance with law, the traditional use of any area of land or water for hunting for food-gathering (otherwise than for purposes of sale) and for ceremonial and religious purposes" (Australian Law Reform Commission, 2010b). An Aboriginal person is defined as "a person of Aboriginal descent who is accepted as a member by a group in the community who claim Aboriginal descent" (Australian Law Reform Commission, 2010a) in this legislation. This paper focuses on kangaroo hunting by non-Aboriginal Australians, unless specifically stated.

14 In the case of Australia, scientific evidence shows it would likely be more beneficial for the Australian landscape to eat feral hunted animals over farmed animals (Adams, 2013; Gressier, 2016). Yet, most meat consumption for humans is still of farmed animals, despite a general consensus amongst Australians that protecting the native Australian nature is a crucial goal (Franklin 2011; Smith 2011; Finch, Murray, Hoy, and Baxter 2014).

15 According to Factiva, the term ‘kangatarianism’ was first used on 12 February 2010 in the Centralian Advocate, the oldest newspaper in the Northern Territory. This information was found searching for the use of the term ‘Kangatar*’ to include the uses of ‘kangatarianism’ and ‘kangatarian’.

16 Anthropologist and sociologist Catie Gressier (2016) mentioned the increase in kangaroo and wallaby consumption and paralleled it to the increase in the consumption of meat from feral animals in Australia driven by an increase of ‘food choices [made] on the basis of perceived environmental impact’ (p. 59).
